# Transgenic *Bt* Rice Does Not Challenge Host Preference of the Target Pest of Rice Leaffolder, *Cnaphalocrocis medinalis* (Lepidoptera: Pyralidae)

**DOI:** 10.1371/journal.pone.0079032

**Published:** 2013-11-11

**Authors:** Xiao Sun, Wen Zhou, Hao Liu, Aijun Zhang, Chao-Ren Ai, Shuang-Shuang Zhou, Chang-Xiang Zhou, Man-Qun Wang

**Affiliations:** 1 Hubei Insect Resources Utilization and Sustainable Pest Management Key Laboratory, College of Plant Science and Technology, Huazhong Agricultural University, Wuhan, P. R. China; 2 Invasive Insect Biocontrol and Behavior Laboratory, United States Department of Agriculture-Agricultural Research Service, Beltsville, Maryland, United States of America; French National Institute for Agricultural Research (INRA), France

## Abstract

**Background:**

Transgenic *Bt* rice line T2A-1 expresses a synthesized *cry2A* gene that shows high resistance to Lepidoptera pests, including *Cnaphalocrocis medinalis* (Guenée) (Lepidoptera: Pyralidae). Plant volatile orientation cues and the physical characteristics of the leaf surface play key roles in host location or host-plant acceptance of phytophagous insects. These volatile compounds and physical traits may become altered in *Bt* rice and it is not known whether this influences the behavior of *C. medinalis* when searching for oviposition sites.

**Results:**

The results of electronic nose analysis showed that the Radar map of *Bt* rice cultivars was analogous to the non- *Bt* rice cultivars at each growing stage. PCA analysis was able to partly discriminate between some of the *Bt* vs. non-*Bt* rice sensors, but could not to separate *Bt* cultivars from non-*Bt* cultivars. The total ion chromatogram between *Bt* and non-*Bt* rice cultivars at the seedling, booting and tillering stages were similar and 25 main compounds were identified by GC-MS. For most compounds, there was no significant difference in compound quantities between *Bt* and non-*Bt* rice cultivars at equivalent growth stages. The densities of the tubercle papicles and the trichomes on the upper and lower surfaces were statistically equal in *Bt* and non-*Bt* rice. The target pest, *C. medinalis*, was attracted to host rice plants, but it could not distinguish between the transgenic and the isogenic rice lines.

**Conclusions:**

There were no significant differences between the *Bt* rice line, T2A-1 and the non*-Bt* rice for volatiles produced or in its physical characteristics and there were no negative impacts on *C. medinalis* oviposition behavior. These results add to the mounting evidence that *Bt* rice has no negative impact on the target insect oviposition behavior.

## Introduction

Transgenic crops that are resistant to insects, due to the expression of *Bacillus thuringiensis* Berliner (*Bt*) genes, have been introduced worldwide and remarkable progress has been achieved [1]–[4]. To date, numerous genotypes of transgenic rice expressing different *Bt* genes have been successfully developed. Laboratory and field investigations have confirmed that *Bt* rice can effectively control the infestation of target Lepidopteran insect pests, such as stem-boring and leaf-folding species [5]–[9]. And a long-term large-scale survey have showed *Bt* crops could enhance biocontrol services in agricultural landscapes [10], however, because there is still a significant ongoing public debate over the social, economic and ecological implications of genetically modified (GM) agriculture [11], [12], the commercial release of *Bt* rice has not been permitted.

Many phytophagous insects use plant volatiles as orientation cues for food plant resources, which are used for nutritional purposes, for mate-location or for depositing their offspring [13]–[18]. The rice leaffolder, *Cnaphalocrocis medinalis* (Guenée) (Lepidoptera: Pyralidae), has been shown to have broad, strong electroantennogram (EAG) responses to individual rice odors [19], [20]. However, it is not known whether these volatile compounds are altered in GM crops. If they have changed, then this may influence the behavior of herbivores searching for oviposition sites natural enemies when foraging [21].

Studies on *Heliothis virescens* (Fabricius) and *Helicoverpa zea* (Boddie) in the Africa suggested that there were no changes in the oviposition behavior of female moths between cotton plant structures after almost 1 decade of widespread *Bt* cotton planting [22], [23]. Van den Berg & van Wyk reported that *Sesamia calamistis* adults did not differentiate between *Bt* and non-*Bt* maize plants during oviposition choice experiments [23]. *Chilo partellus* and *Sesamia calamistis* moths could not discriminate between *Bt* and non-*Bt* maize plants with regards to egg laying [24] and uninfested *Bt* and non-*Bt* maize were similarly attractive to females of the larval parasitoids: *Cotesia flavipes* and *C. sesamiae* [25]. However, it has been reported that there were significant quantitative differences in volatile emissions between *Bt* and non-*Bt* plants, although *C. marginiventris* and *Microplitis rufiventris* could not distinguish between the odors of a *Bt* maize cultivar and its near-isogenic line [26]. *H. armigera* hatching larvae have been shown to preferentially seek structures with lower Cry protein levels when selecting plants to feed on [27]. In addition, the olfactory responses of the parasitoid *Cotesia marginiventris* were shown to be weaker toward host (*Spodoptera frugiperda*) frass derived from Bt maize compared to frass derived from conventional maize [28].

Most insect-plant interactions occur on the leaf surface. The physical and/or chemical characteristics of the leaf surface play key roles in host-plant acceptance by phytophagous insects. Any changes to the physical and/or chemical characteristics of the leaf surfaces of transgenic crops, due to the insertion of exotic genes, may influence the search for or the acceptance of host plants by herbivore insects [21]. Significant phenotypic changes induced by genetic modification are not without precedent. Xue showed that three cotton lines were quite different from each other in the densities of certain kinds of covering trichomes [29] and the production of lignin, a major structural component of plants, increased in vascular tissues by 33–97% in *Bt* maize compared to non-*Bt* isolines [30].

The transgenic *Bt* rice line, T2A-1, expresses a synthesized *cry2A* gene and has a high resistance to Lepidoptera pests [7], [8], including *Cnaphalocrocis medinalis* [31]. It has been reported that T2A-1 has no significant effects on the occurrence of four predatory arthropod natural enemies (*Ummeliata insecticeps*, *Paegerus fuscipes*, *Hylyphantes graminicola*, *Pardosa pseudoannulata*) of *C. medinalis* [31] and previous studies have reported no significant adverse effects on the population dynamics of three plant hoppers (*Nilaparvata lugens*, *Sogatella furcifera* and *Laodelphax striatellus*) and their predators (*Cyrtorhinus lividipennis*, *Pirata subpiraticus* and *Theridium octomaculatum*) [32]. But it is not clear whether *Bt* genetic engineering in rice may have an effect on phenotypic changes (e.g. densities coverings and volatile emissions) which could cause unintended pleiotropic effects in the environment. The aim of the study was to investigate whether *Bt* genetic engineering in rice may affect volatile emissions of rice plants as well as olfactory- & physically-mediated host location behavior of *C. medinalis*.

## Materials and Methods

### Plant Growth

The rice varieties used in this study were the transgenic *Bt* rice line, T2A-1 and the non-transgenic parental *indica* rice line, Minghui 63 (MH63). T2A-1 expresses a synthesized *cry2A* gene which was driven by the maize *ubiquitin* promoter and introduced into the elite indica rice restorer line Minghui 63 by *Agrobacterium*-mediated transformation. All the rice lines were gifted by Lin Yong-Jun, National Key Laboratory of Crop Genetic Improvement, Wuhan, China. The rice seeds were sown in a greenhouse under 26±4°C, a 16 h light:8 h dark cycle and 60–90% relative humidity. After 20–25 d, the seedlings were transplanted into clay pots (20 cm (diam)×30 cm (height)) and each pot contained one plant. The plants were watered daily, and each pot was supplied with 10 ml of nutrient solution (Ca(NO_3_)_2_·4H_2_O, 0.5 g/l; K(NO_3_)_2_·4H_2_O, 0.125 g/l; MgSO_4_·7H_2_O, 0.125 g/l; K_2_HPO_4_, 0.125 g/l and FeCl_2_, 0.005 g/l) every 3 d. The pots were divided into three groups: plants representing the seedling stage, 25–30 d after potting for the tillering stage and 20–25 d after potting for the booting stage. Planting continued at regular intervals so that there were enough plants of a suitable age available for all the experiments.

### Electronic Nose Measurement

The electronic nose, FOX 4000 (Alpha MOS, France), was used in this study. It was equipped with 18 different thermo-regulated metal oxide semiconductor sensors that were sensitive to different classes of chemical compounds (Table S1). The equipment used clean air (through an activated charcoal filter) as a reference gas. For each sample, three seedlings or three flag leaves at the tillering/booting stage were placed in 100 ml Pyrex vials equipped with a pierceable silicon/Teflon cap. Measurements were performed in dynamic headspace mode by extracting the headspace through the 0.45 µm syringe filter outlet in order to prevent environmental contamination. Each measurement cycle consisted of: exposure of the sensors to clean filtered ambient air, a sensor measurement period of 120 s, exposure of the sensors to the sample headspace for 120 s and finally, sensor exposure to filtered ambient air for 360 s for baseline recovery before the next analysis. The experimental conditions adapted from Camurati *et al* were used [33], and measurement was repeated six times for each sample. In order to perform data statistical correlation, e-nose measurements were performed at the same time when volatile samples were collected for Gas chromatography/mass spectrometry (GC-MS) analyses.

### Collection, Isolation and Identification of Volatile Compounds

The seedlings and potted plants (1 or 3 per pot) were washed with running water to remove soil. The treated plants were placed in water-filled conical flasks, which were wrapped with tinfoil. The plants were then placed separately into four glass containers (20 cm (diam)×50 cm (height)) and connected to Super Q (200 mg each, Alltech Associates, Inc. Deerfield, Illinois) traps (15 cm L×0.6 cm OD) [34]. Air was filtered using three adsorbent traps (20 cm×3 cm OD): 1) charcoal (Activated Carbon, 6–14 mesh, Fisher Scientific), 2) 5A molecular sieves (beads, 8–12 mesh, Sigma-Fluka), and 3) silica gel Rubin (Drying agent free of metal salts* silica gel, Sigma-Fluka) before being pulled through the apparatus with a vacuum pump. The flow rate was controlled at 0.4 L/min, and volatile collection was conducted at 25±1°C for 24 hours (Figure S1). The airborne volatiles were eluted by percolating each Super Q trap with glass-distilled dichlormethane (0.5 ml/container), and the resultant solutions were concentrated using nitrogen to a volume of about 100 µl. Exactly 200 ng of nonyl acetate (Sigma, Buchs, Switzerland) in 10 µl of dichlormethane was added to the samples as internal standards and the solutions were then stored at –40°C. Collections were replicated five times for each cultivar.

GC/MS analysis was performed on DSQ II instrument (Thermo Fisher Scientific, USA) with an automated on-column injection system. A 1 µl aliquot from each sample was injected onto a HP-5 capillary column (30 m, 0.25 mm i.d. and with a 0.25 mm film thickness, Alltech, Deerfield, IL, USA) in splitless mode. Helium (24 cm/s) was used as the carrier gas. After injection, the oven temperature was maintained at 40°C for 2 min, increased to 250°C at 6°C/min and then held at 250°C for 2 min. The detector signal was processed using Hewlett Packard (HP) GC Chemstation software.

Initial identification of volatile compounds was based on the Wiley MS library data base matching and comparisons of retention times with those from previous studies [35]. The identities of potential candidate compounds and were confirmed by GC retention times and MS spectra of the standard samples. For the quantitative purpose, 1 µl aliquot was injected in pulsed splitless mode into a HP GC equipped with a flame ionization detector, using the same column and temperature program as above. Total volatile contents were calculated based on their peak areas when compared to those of the internal standards.

### Trichome and Tubercle Papicle Density

Flag leaves from plants at the booting stage were collected. Measurements were undertaken using a scanning electron microscope (SEM) (Tokyo, Japan) depending on the number of trichomes and tubercle papicles on the upper and lower surfaces [29]. Thirty samples were counted per treatment and the subsequent data is presented in terms of trichome and tubercle papicle density per 0.1 mm^2^.

### Bioassays

*Cnaphalocrocis medinalis* pupae were collected from an experimental plot in Wu’xue county of Hubei province in China (115°33′E; 29°51′N) in 2012. The locations sampled were not privately owned or protected in any way, and this field study did not involve endangered or protected species. Sexed pupae were kept inside culture dishes (20 cm diameter) in an environmental chamber (25±1°C, 75±5% RH and 16 L:8 D photoperiod) until the moths emerged. In order to assess oviposition preference, ten doubles of newly emerged females and male *C. medinalis* were released in a cage covered with a cotton mesh (70×70×100 cm) that contained four potted plants (two MH-63 and two T2A-1 plants) at the booting stage and placed in a diagonal formation (Figure S2).

The assay consisted of 30 replicates and was carried out in an outdoor environment under high humidity conditions (Figure S2). The position of the *Bt* and non-*Bt* rice in the cages was alternately changed in the different replicates. The plants were exposed for oviposition for 6 d and then the number of eggs laid was recorded.

### Data Analysis

The complex data sets created by e-nose analysis were submitted to Principal Component Analysis (PCA) using a projection method that allowed an easy visualization of the information contained in the data sets and also permitted dimensional reduction [36]. Two-way ANOVA was used to analyze the differences between cultivars and stages, together with their interactions, on the measured indexes of relative abundance (*P*-value). A paired-sample *t*-test was used to analyze the differences in the quantity of volatiles, trichome/tubercle papicle density and *C. medinalis* oviposition performance between MH63 and T2A-1. The data were analyzed statistically using SPSS 20 for Windows software.

## Results

### Electronic Nose Analysis

**Figure 1:**
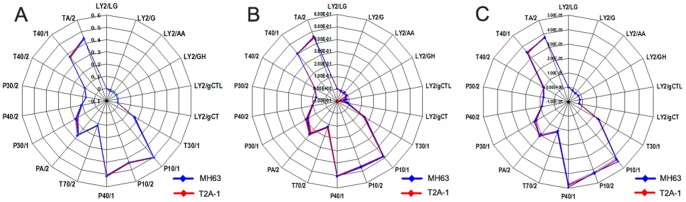
Radar map, created using an electronic nose, of rice leaves from *Bt* and non-*Bt* rice cultivars: (A) seedling stage(S); (B) tillering stage (T); (C) booting stage (B).

The response values for each sensor are shown in Figure 1. These graphs were constructed using the changes in relative resistance and represented the peak height of each sensor as a radial vector. The sensor responses were highly reproducible and the outline of *Bt* rice cultivars was analogous to the non-*Bt* rice cultivars. Two-way ANOVA analysis showed that there were no obvious changes in relative abundance for each sensor when comparing the two cultivars (*P*>0.05) and cultivar×stage did not affect relative abundance either (*P*>0.05), but growth stage did significantly affect sensor relative abundance (*P*<0.05) (Table 1).

**Table 1:** Two-way ANOVAs of the effects of cultivar, stage and their interactions on the measured indexes of relative abundance (*P*-value).

Alpha MOS Sensor Type	Cultivar	Stage	Cultivar×Stage
LY2/LG	0.481	0.534	0.281
LY2/G	0.900	<0.0001	0.425
LY2/AA	0.633	<0.0001	0.352
LY2/GH	0.825	<0.0001	0.071
LY2/gCTL	0.817	<0.0001	0.974
LY2/gCT	0.236	<0.0001	0.594
T30/1	0.917	<0.0001	0.261
P10/1	0.529	<0.0001	0.713
P10/2	0.433	<0.0001	0.730
P40/1	0.486	<0.0001	0.671
T70/2	0.547	<0.0001	0.794
PA/2	0.928	<0.0001	0.911
P30/1	0.851	<0.0001	0.322
P40/2	0.813	<0.0001	0.524
P30/2	0.466	<0.0001	0.723
T40/2	0.766	<0.0001	0.671
T40/1	0.506	<0.0001	0.468
TA/2	0.409	<0.0001	0.573

**Figure 2:**
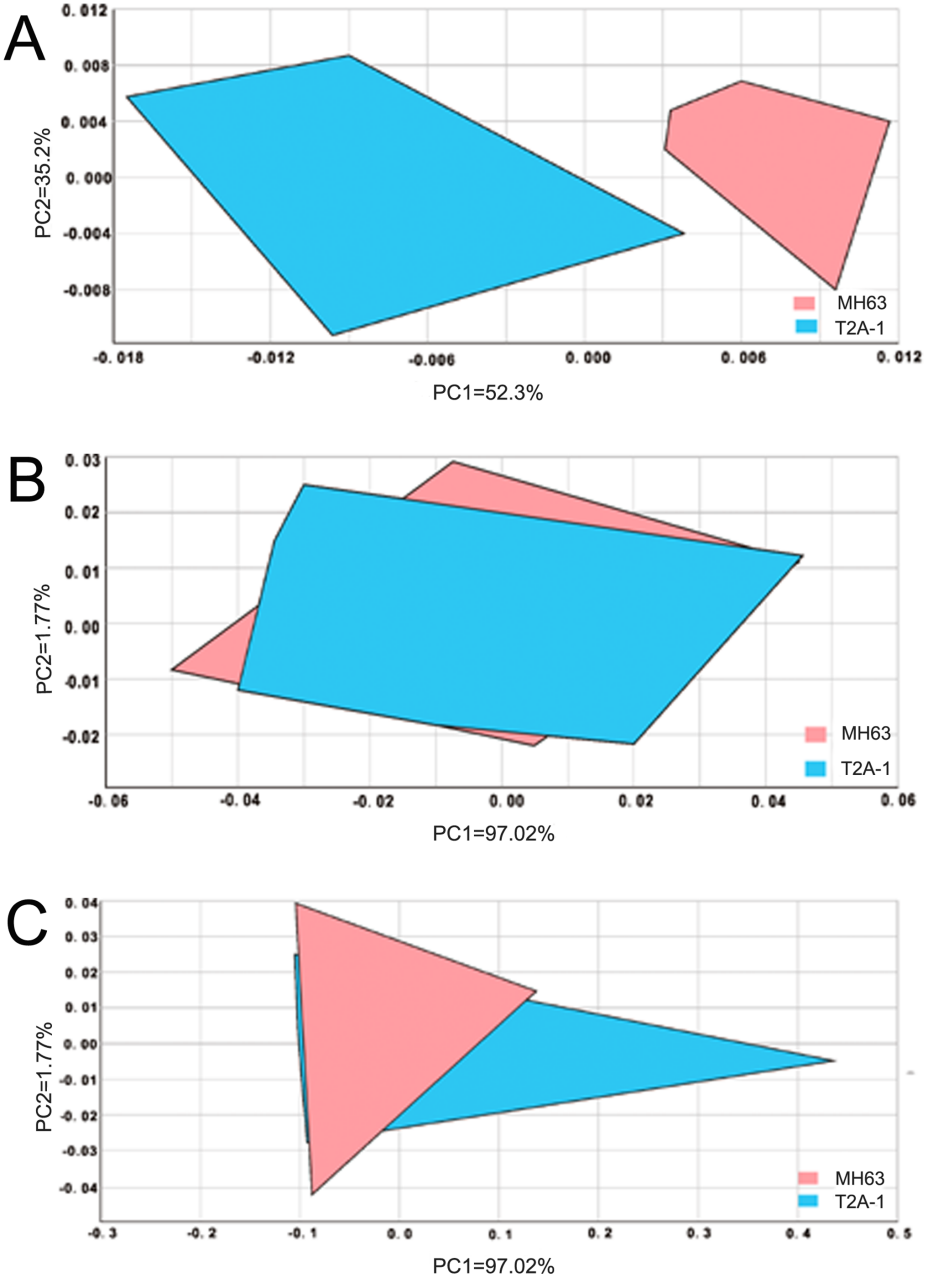
Score plots of PC1 vs. PC2 for the PCAs of leaves from *Bt* and non-*Bt* rice cultivars analyzed using an electronic nose: (A) seedling stage (S); (B) tillering stage (T); (C) booting stage (B).

The PCA analysis results are shown in Figure 2, which exhibited the results for principal component1 (PC1) and principal component2 (PC2) on a two-dimensional plane. PCA is viewed as a linear combinatorial method and enabled the complexity of the data set to be reduced. PCA was performed in order to describe the volatile changes seen during the different stages for *Bt* and non-*Bt* rice cultivars. At the seedling stage, the first principal component, PC1, explained 52.30% of the total variation, while 35.21% of the total variance was explained by PC2, with a distance of 0.01 (Figure 2A). At the tillering stage, PC1 explained 66.33% of the variation, while PC2 explained 20.89%, with a distance of 0.02 (Figure 2B). At the booting stage, the values were 97.02% for PC1 and 1.77% for PC2, with a distance of 0.04 (Figure 2C). The distances indicated that there was a low discrimination power between the components of the non-*Bt* and *Bt* samples at all three growth stages. This method was able to partially discriminate between the *Bt* and non-*Bt* rice cultivars for some of the sensors but was not able to separate out the *Bt* cultivar from the non-*Bt* cultivar.

### Volatile Compounds Emitted from Rice

**Figure 3:**
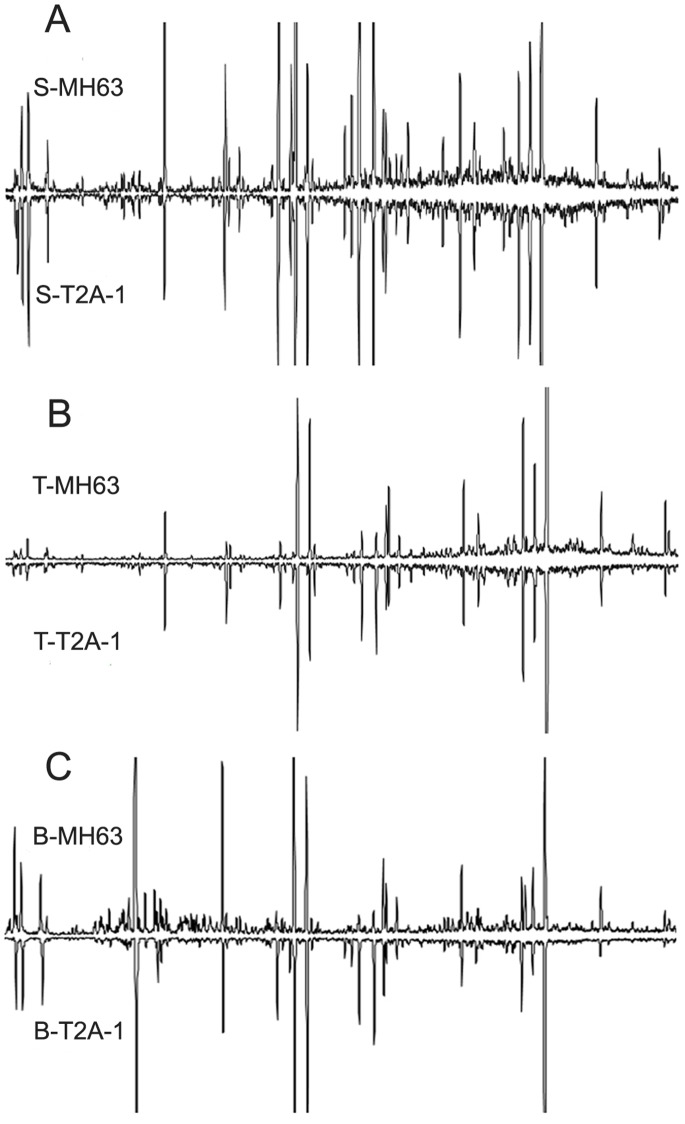
Gas chromatography-mass spectrometry (GC-MS) profiles of representative solvent extracts of *Bt* and non-*Bt* rice cultivars: (A) seedling stage (S); (B) tillering stage (T); (C) booting stage (B).

The total ion chromatogram between *Bt* and non- *Bt* rice cultivars at the seedling, booting and tillering stages were similar (Figure 3) and 25 main compounds, belonging to the following chemical classes: alkanes, terpenoids, carbonyls, hydroxyl and carbonyl compounds, benzenes, aromatic compounds and heterocyclil compounds, were identified by GC-MS (Table 2). The alkanes, such as: dodecane, tridecane, tetradecane, pentadecane, hexadecane, eicosane, heneicosane and tetracosane, were the most abundant class of compounds, whereas the major components identified were alkanes. Naphthalene and dodecane were the predominant components identified at all three growth stages.

**Table 2:** Volatiles released at the seedling (S), tillering (T) and booting (B) stages by *Bt* and non-*Bt* rice.

Volatile	S-T2A-1	S-MH63	T-T2A-1	T-MH63	B-T2A-1	B-MH63
Alkanes						
Dodecane	1.95±0.27	1.51±0.07^ns^	3.50±0.54	4.12±0.26^ns^	3.82±0.96	5.06±1.66^ns^
Tridecane	1.94±0.20	1.82±0.15^ns^	1.10±0.44	1.12±0.25^ns^	0.99±0.17	1.00±0.73^ns^
Heptylcyclohexane	0.25±0.69	0.24±0.37^ns^	0.19±0.26	0.17±0.20^ns^	0.15±0.12	0.13±0.13^ns^
Tetradecane	1.96±0.14	1.63±0.93^ns^	1.97±0.20	1.82±0.15^ns^	1.43±0.49	1.31±0.25^ns^
Pentadecane	2.16±0.18	1.87±0.04^ns^	2.76±0.97	2.83±0.19^ns^	1.31±0.64	1.29±0.31^ns^
Hexadecane	1.73±0.23	1.77±0.13^ns^	1.66±0.11	1.90±0.18^ns^	0.95±0.87	1.02±0.19^ns^
Eicosane	1.53±0.15	1.71±0.10^ns^	1.29±0.18	1.68±0.19^ns^	0.80±0.26	0.72±0.18^ns^
Heneicosane	0.66±0.25	0.66±0.11^ns^	1.40±0.18	1.67±0.19^ns^	0.37±0.64	0.56±0.28^ns^
Tetracosane	0.24±0.36	0.21±0.23^ns^	0.39±0.12	0.44±0.91^ns^	0.17±0.15	0.16±0.34^ns^
Terpenoids						
Longifolene-(V4)	0.53±0.08	0.49±0.07^ns^	0.40±0.10	0.41±0.07^ns^	0.23±0.10	0.30±0.05^ns^
Cedrene	1.26±0.12	0.95±0.12^ns^	1.42±0.94	1.35±0.11^ns^	0.72±0.27	0.31±0.54^ns^
4-Carene	0.17±0.31	0.17±0.03^ns^	0.16±0.12	0.17±0.21^ns^	0.15±0.09	0.15±0.02^ns^
à-Pinene	0.20±0.15	0.17±0.26^ns^	0.22±0.41	0.19±0.19^ns^	0.17±0.16	0.16±0.16^ns^
D-Limonene	1.75±0.15	1.99±0.37^ns^	1.74±0.32	1.88±0.30^ns^	0.47±0.25	0.44±0.44^ns^
Carbonyls						
Tetradecanal	0.48±0.67	0.49±0.68^ns^	0.18±0.14	0.15±0.13^ns^	0.09±0.31	0.17±0.12^ns^
Nonanal	0.56±0.35	0.54±0.24^ns^	0.68±0.067	0.65±0.68^ns^	0.28±0.30	0.30±0.36^ns^
2-Hexanone	0.20±0.02	0.22±0.47^ns^	0.18±0.58	0.13±0.13^ns^	0.07±0.01	0.07±0.02^ns^
Hydroxyl and carbonyl compound						
Cedrol	0.33±0.12	0.35±0.23^ns^	0.34±0.47	0.35±0.45^ns^	0.18±0.06	0.20±0.35^ns^
3-Hexanol	0.81±0.18	0.95±0.18^ns^	0.25±0.18	0.26±0.43^ns^	0.28±0.41	0.31±0.78^ns^
(Z)3-Hexen-1-ol	1.11±0.64	1.17±0.22^ns^	1.20±0.14	1.54±0.20^ns^	0.10±0.01	0.14±0.24^ns^
Benzene						
Ethylbenzene	1.17±0.10	0.80±0.07*	0.35±0.04	0.36±0.23^ns^	1.89±0.31	1.87±0.47^ns^
o-Xylene	1.10±0.22	0.75±0.04^ns^	0.30±0.06	0.45±0.06^ns^	1.27±0.11	1.34±0.25^ns^
Biphenyl	0.39±0.08	0.41±0.06^ns^	0.40±0.92	0.50±0.95^ns^	0.32±0.60	0.27±0.06^ns^
Aromatic compounds						
Indane	0.10±0.01	0.08±0.01^ns^	0.06±0.02	0.09±0.02^ns^	0.13±0.02	0.14±0.04^ns^
Heterocyclil compound						
Naphthalene	7.40±0.30	6.47±0.64^ns^	5.03±0.35	5.20±0.61^ns^	5.24±1.12	6.12±0.89^ns^

Note: paired values with an asterisk indicate that there is an significant difference between *Bt* (T2A-1) and non-*Bt* (MH63) rice (*P*<0.05) and ^ns^ indicates there are no significant differences between *Bt* (T2A-1) and non-*Bt* (MH63) rice at the seedling stage (S), tillering stage (T) and booting stage (B), respectively.

For each compound, with the exception of the ethylbenzene content in *Bt* rice, which was higher than that in non-*Bt* rice *(P*>0.05), there were no significant quantitative differences between *Bt* and non-*Bt* rice cultivars at the same growth stage (*P*>0.05). However, within each rice cultivar, the quantity of some volatiles changed as the rice grew and, both rice cultivars showed the same trend, which had three distinct patterns: first there was an uptrend for some compounds, such as dodecane, then there was a downtrend for some compounds, such as 2-hexanone, 3-hexanol, D-limonene, tridecane, tetradecanal, heptylcyclohexane, longifolene-(V4) and eicosane, and finally the trend was flat for some compounds, such as 4-carene, à-pinene, and naphthalene (Table 2).

### Trichome and Tubercle Papicle Density

**Figure 4:**
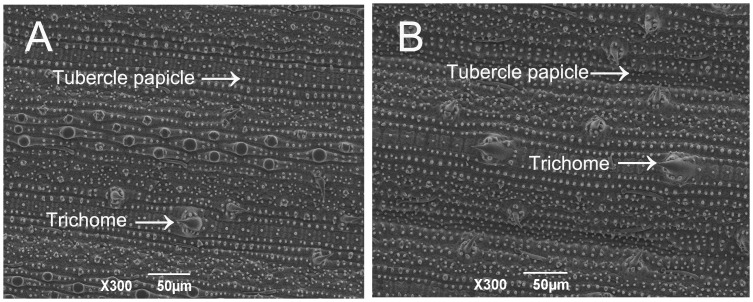
The morphology of trichomes and tubercle papicles on the surfaces of rice leaves: (A) upper surfaces; (B) lower surfaces.

**Figure 5:**
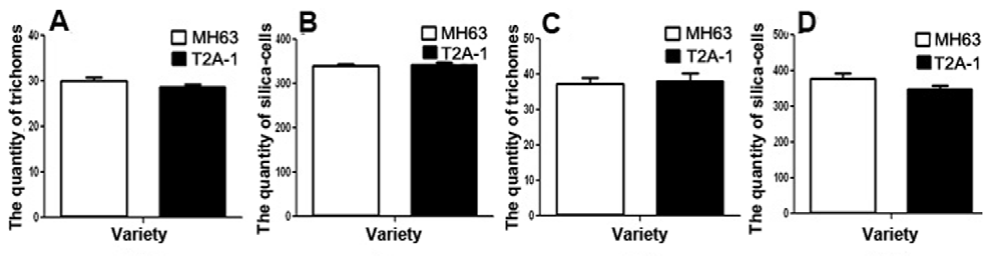
Density of trichomes and tubercle papicles on the surfaces of rice leaves: (A) trichomes on the upper surfaces; (B) tubercle papicles on the upper surfaces; (C): trichomes on the lower surfaces; (D) tubercle papicles on the lower surfaces.

Under the SEM, tubercle papicles were found to be widespread across the leaf surfaces of *Bt* rice and non-*Bt* rice lines and the trichomes were located along the veins on both surfaces of the leaf (Figure 4). There were no significant differences in the total density of the different types of trichomes covering the upper surface (*t* = 1.5105; *P* = 0.1363), the lower surface (*t* = 0.2569; *P* = 0.7981) or between the two rice lines. There were also no significant differences between the two rice lines for tubercle papicles on the upper surface (*t* = 0.3271; *P* = 0.7448)) or on the lower leaf surface (*t* = 1.7119; *P* = 0.0931) (Figure 5).

### Target Pest Performance

**Figure 6:**
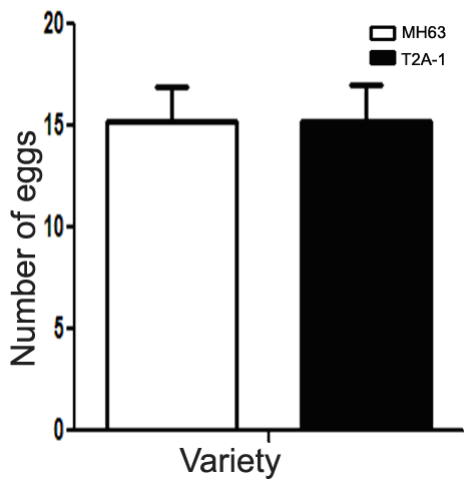
The number of eggs (mean ± SE) on *Bt* and non-*Bt* rice.

The average number of eggs (mean ± SE) per *Bt* and non-*Bt* rice plant was 15.2 and 15.1, respectively, and there were no significant differences between the two rice lines (*t* = 0.0219; *P* = 0.9826) (Figure 6).

## Discussion

Concern over the environmental safety of *Bt* crops has led to a large number of studies that have investigated the potential effect of these crops on non-target phytophagous insects and their natural enemies. In general, significant negative effects or not on natural enemies due to *Bt*-transgenic rice have been observed, as measured by fitness indicators, population density and dynamics and biodiversity indices [37]–[42]. However, little attention has been paid to the potential changes in rice plant volatiles due to the genetic modification of rice plants, which could affect the insect host location process.

The electronic nose was based on the principal of gas chromatography and an array of solid-state sensors that are non-selectively sensitive to the relevant chemicals and the responses of which reflect the chemical information contained in the sample. This detection scheme is, in many respects, similar to natural olfaction where hundreds of different receptors can distinguish between tens of thousands of different odors [43]. FOX 4000 electronic noses have been applied in a number of different fields and have identified and classified many different samples [43]–[46]. In this study, for the first time, the FOX 4000 has been used to compare *Bt* and non-*Bt* rice. Both the Radar map analysis and the PCA results showed that there were no significant differences in volatiles between the *Bt* and non-*Bt* rice cultivars, whereas growth stage could affect volatile contents in the two cultivars. However, it was hard to discriminate between the *Bt* and non-*Bt* rice varieties on the Radar maps and by PCA after using the electronic nose.

Overall, the types of volatiles present in *Bt* and non-*Bt* rice identified by GC-MS were similar and in good agreement with those previously reported in non-*Bt* rice cultivars using different extraction methods [47]–[50]. There were no significant differences for almost compounds between the two rice lines at all three growth stages. This suggested that genetic modification did not appear to alter the volatile chemical profile of rice. The same results have been found in *Bt* maize, where genetic modification did not alter the volatile chemical profile of undamaged maize [51]. Furthermore, *Bt* maize did not alter the aphid–parasitoid associations and had no effect on the aphid parasitism and hyperparasitism rates [52]. However, ethylbenzene at the seeding stage where the results showed a significant difference between *Bt* and non-*Bt* rice. Ethylbenzene is in a range of plants such as green coffee [53], *Olea europaea* [54] and *Coffea Arabica* [55]. Ethylbenzene emitted by the leaves and half-ripe olives was significantly attractive to *Dacus oleae*, while as an oviposition weak activants of *D. oleae* [54]. In the olfactometer bioassay, a pest of plants *Hypothenemus hampei* showed a significant response to ethylbenzene emitted by the *Coffea Arabica* [55]. While the correlation of ethylbenzene emitted by rice to the rice pests or natural enemies are not clear. Differences between *Bt* and non-*Bt* plants, including changes in the blend of plant volatiles emitted, have been found and undamaged *Bt* cotton plants have been shown to emit unique compounds and different proportions of typical compounds when compared to non-*Bt* cotton [56]. The HD-non *Bt* cultivar has been found to release a higher quantity of volatile compounds compared to HD-*Bt* maize [26] and transgenic *Bt* (expressing the *cry1Ac* endotoxin gene) oilseed rape plants affect the emissions of DMNT and (E,E)-alpha-farnesene after herbivore damage [57]. The headspace volatiles of two representative cultivars of transgenic scab resistant apple plants differed quantitatively for four terpenes and an aromatic compound [58]. The data available to date, relating to *Bt* transgenes and induced volatile release by crop plants implied that the diversity of effects by genetic modification could be obtained in different plant species.

In addition, leaf surface physical characteristics can influence the searching behaviors of insects [59]. Physical factors on the leaf surface are mainly exhibited as the density of trichomes and tubercle papicles. There are two primary types of epidermal outgrowths, i.e. covering trichomes and capitate or glandular trichomes [60], [61]. The results of this study showed that the covering trichomes and tubercle papicle densities were similar in *Bt* and non-*Bt* rice lines. Studies have also shown that there were no significant differences in the physical profiles of *Bt* and non-*Bt* arabidopsis plants [62] and cotton plants [56].

The bioassay results indicated that *C. medinalis* oviposition was similar on the *Bt* rice lines and the regular rice line. The target pest, *C. medinalis*, was attracted to the host rice plants, but couldn’t distinguish between the transgenic and the isogenic rice lines. As far as can be ascertained, this is the first study that describes the changes in volatile and physical characteristics introduced by the *Bt* gene in rice and this study has been the one of the very few that has focused on the target pest behavioral response to *Bt* plants. These results add to the mounting evidence that *Bt* rice has no negative impact on target insect behavior. Plant volatile emissions are likely to be affected by herbivore damage, which could have an effect of on plant-herbivore-parasitoid tritrophic relationships. The herbivore-induced volatile emissions from *Bt* rice plants compared to those from a non-transformed isoline will be studied further in the future.

## Supporting Information

Figure S1 **The headspace collection of both Bt and non-Bt rice.**
(TIF)

Figure S2 **Oviposition preference bioassays of** ***Cnaphalocrocis medinalis*** **: (A)** Rice plants and target pests were covered with net in order to prevent the pests from flying away; **(B)** Experiments were conducted in humid and warm conditions.
(TIF)

Table S1 **Sensor sensitivity of eighteen individual sensors within the sensor array of the FOX 4000 e-nose.**
(TIF)
