# Supplementary figures and images for: Transgenic Bt Rice Does Not Challenge Host Preference of the Target Pest of Rice Leaffolder, Cnaphalocrocis medinalis (Lepidoptera: Pyralidae)
Source: PLoS One. 2013 Nov 11;8(11):e79032. doi: 10.1371/journal.pone.0079032 (PMC3823965; doi:10.1371/journal.pone.0079032)

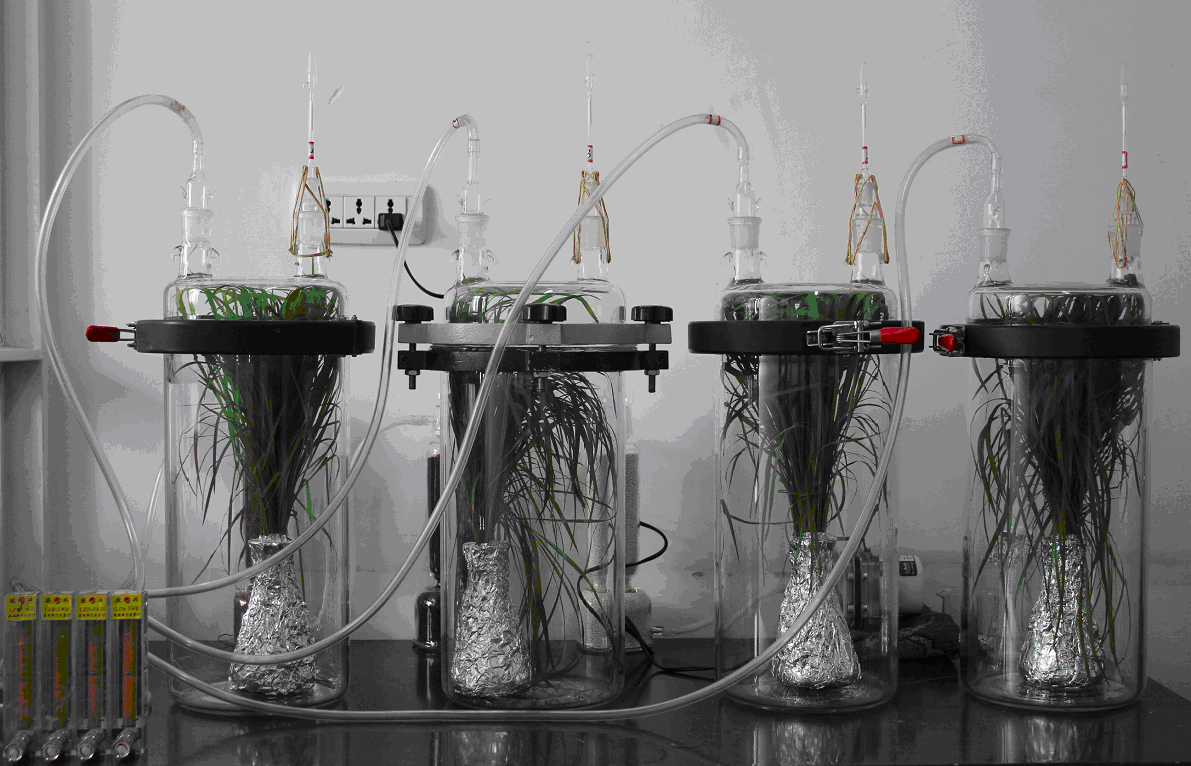

Supplement: Figure S1 — The headspace collection of both Bt and non-Bt rice. (TIF) [file pone.0079032.s001.tif]

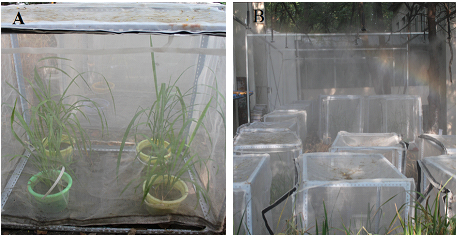

Supplement: Figure S2 — Oviposition preference bioassays of Cnaphalocrocis medinalis : (A) Rice plants and target pests were covered with net in order to prevent the pests from flying away; (B) Experiments were conducted in humid and warm conditions. (TIF) [file pone.0079032.s002.tif]

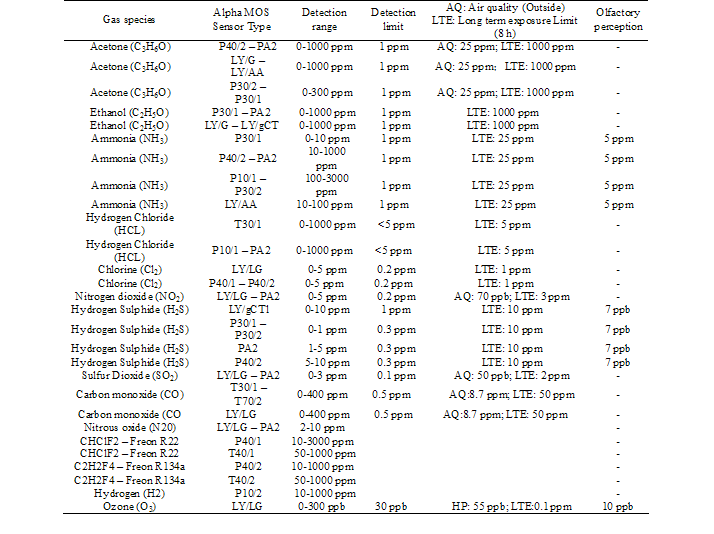

Supplement: Table S1 — Sensor sensitivity of eighteen individual sensors within the sensor array of the FOX 4000 e-nose. (TIF) [file pone.0079032.s003.tif]
